# A Review of Infections After Hematopoietic Cell Transplantation Requiring PICU Care: Transplant Timeline Is Key

**DOI:** 10.3389/fped.2021.634449

**Published:** 2021-07-27

**Authors:** Asmaa Ferdjallah, Jo-Anne H. Young, Margaret L. MacMillan

**Affiliations:** ^1^Department of Pediatrics, Division of Blood and Marrow Transplantation and Cellular Therapy, University of Minnesota, Minneapolis, MN, United States; ^2^Department of Medicine, Division of Infectious Disease and International Medicine, Program in Transplant Infectious Disease, University of Minnesota, Minneapolis, MN, United States

**Keywords:** hematopoietic stem cell transplantation, pediatric, immunosuppressed, sepsis, opportunistic infection

## Abstract

Despite major advances in antimicrobial prophylaxis and therapy, opportunistic infections remain a major cause of morbidity and mortality after pediatric hematopoietic cell transplant (HCT). Risk factors associated with the development of opportunistic infections include the patient's underlying disease, previous infection history, co-morbidities, source of the donor graft, preparative therapy prior to the graft infusion, immunosuppressive agents, early and late toxicities after transplant, and graft-vs.-host disease (GVHD). Additionally, the risk for and type of infection changes throughout the HCT course and is greatly influenced by the degree and duration of immunosuppression of the HCT recipient. Hematopoietic cell transplant recipients are at high risk for rapid clinical decompensation from infections. The pediatric intensivist must remain abreast of the status of the timeline from HCT to understand the risk for different infections. This review will serve to highlight the infection risks over the year-long course of the HCT process and to provide key clinical considerations for the pediatric intensivist by presenting a series of hypothetical HCT cases.

## Introduction

Hematopoietic cell transplant (HCT) is a potential cure for many malignant and non-malignant diseases (1). As the indications for HCT broaden to include many genetic and inherited metabolic disorders, and the use of other cellular therapies increases, pediatric patients will undergo HCT at increasing volumes (2, 3).

In the pediatric intensive care unit (PICU), sepsis and infectious complications are common causes for admission after HCT (4). In a University of California study of 1,782 pediatric HCT patients admitted to the PICU, infection was documented in 45.7% of admissions, with 22.2% mortality (5). Hematopoietic cell transplant recipients were found to more likely present in septic shock and/or with respiratory failure than non-HCT PICU patients. Infections remain the leading immediate cause of mortality in HCT patients in the PICU (5). An Italian study of 496 children admitted to the PICU following HCT reported a mortality rate of 30–40% (6).

Hematopoietic cell transplant patients are at extraordinarily high risk for opportunistic infections for at least a year after transplant depending on several factors including underlying disease, donor graft source, and conditioning regimen among others; these factors will be discussed in further detail throughout this article (7–10). The risk for specific infections greatly varies during this timeline and depends upon several factors, particularly the state of the patient's immune system, which will not return to normal for at least a year after HCT (7). It is important to have a keen understanding of the various stages of HCT, associated risk factors for infections, and the state of the immune system throughout the HCT timeline, to successfully treat opportunistic infections in HCT patients.

## Hematopoietic Cell Transplant

The goal of a HCT is to eliminate a patient's native bone marrow (BM) cells and to replace them with healthy donor cells (11). These native stem cells may contain a genetic mutation that is driving a certain disease state, or may be defective resulting in BM failure, or a neoplasm that cannot be destroyed with conventional chemotherapy or radiation (11). To destroy these errant stem cells, high dose chemotherapy and often radiation, are administered to ablate the BM to make space for new stem cells to begin the process of renewed hematopoiesis (12). With malignant diseases, chemotherapy and radiation, “the preparative regimen,” also serves the purpose of killing any remaining residual cancer cells that may be present in the BM (13, 14). Donor cells are taken from the BM, which is the origin of stem cell production and differentiation, and this process is also referred to as a bone marrow transplant (BMT) (15). Alternatively, donor cells can be mobilized by administering medications to the donor and subsequently collecting the stem cells from the peripheral blood (a peripheral blood stem cell transplantation) (16). Donor stem cells are considered *allogeneic*, meaning they originate from another individual and not the patient (17). It is important to understand that the very nature of an allogenic transplant necessitates the use of immunosuppression for a short time to allow for the immunologic establishment of the new stem cells (17). For some conditions, the cure can be achieved with high dose chemotherapy followed by the infusion of a patient's own, previously collected, stem cells (11). This is termed an *autologous* HCT (11).

While relapsed, recurrent, and high-risk malignancies are the most common indications for HCT, the expanding list of conditions treated by HCT is diverse (18). Other indications for HCT include hemoglobinopathies, immune dysregulation, or deficiencies, BM failure syndromes, and inherited congenital metabolic disorders (18, 19). Some of these patients, such as those with BM failure syndromes or inherited congenital syndromes, will not have experienced extensive treatment for their disease before transplant (18). However, others, particularly those with certain types of aplastic anemia and malignancy, will have been heavily treated with chemotherapeutic agents and/or immunosuppressive drugs even prior to transplant (20). This is especially the case for patients with acute leukemia who may have received multiple courses of profoundly immunosuppressive therapy before transplant to achieve remission (21). Bone marrow failure patients may have spent weeks to years with varying states of neutropenia and so their period of high-risk status is longer than other patients (22). Additionally, patients with prolonged neutropenia may have had previously treated opportunistic infections prior to HCT (23). Providers should note the individual degree of neutropenia before HCT while caring for these patients for context when choosing diagnostic tests and empiric treatment agents for infections after HCT (23).

## Preparative Regimen

The preparative regimen is typically administered for 5–7 days prior to the transplant day (referred to as Day 0), depending on the underlying disease state (24). It includes high-dose chemotherapy with or without radiation (12). Immunosuppression is also started a few days prior to transplant for allogeneic HCT to allow for therapeutic drug serum levels as new stem cells establish themselves (25). During this early critical period, immunosuppression is key to prevent donor T lymphocytes from recognizing recipient cell antigens as foreign and attacking various organ systems (26). It must be noted however that although this immunosuppression is necessary, it contributes to delayed immune recovery following HCT (25).

Although anemia, lymphopenia, neutropenia, and thrombocytopenia usually become more severe as the transplant day nears and the cumulative effects of the preparative regimen are manifested, the patient's blood counts may not necessarily drop to zero by transplant day (27, 28). Despite this fact, this period places patients at high risk for spontaneous infection as these residual neutrophils do not function properly because of profound immune suppression (29). Existing portals of entry such as mucosal breakdown from mucositis and skin breaches from the presence of a central venous access line are risk factors for infection (29).

The preparative characteristic that is most predictive of risk of infection is its intensity which dictates the severity of tissue damage including mucositis and the duration of pancytopenia that follows (12). Patients receive various degrees of marrow ablating chemotherapy as determined by their underlying disease and comorbidities (12). Most pediatric patients with leukemia will receive a myeloablative regimen—which destroys the BM's ability to regenerate stem cells and necessitates a replacement by newly donated stem cells (14). As such, myeloablative approaches (which may also include total body irradiation) lead to prolonged periods of impaired mucosal barrier and neutropenia, leaving patients at risk for neutropenia-related opportunistic infection for the initial 3–6 weeks following transplantation (29). After neutrophils engraft and become stronger in number, the persisting immune deficit is lymphopenia. Lymphopenia gradually improves over the first year following transplantation.

**Figure 1:**
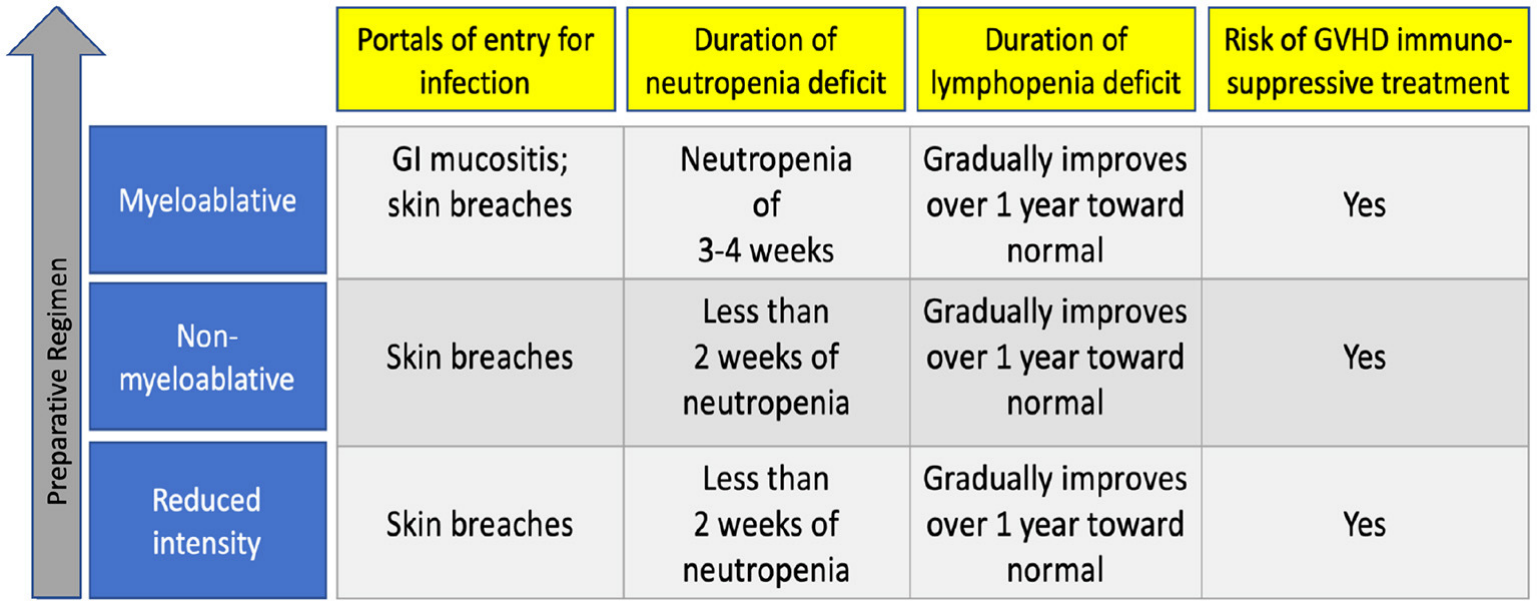
Factors associated with infections by preparative therapy intensity.

Other preparative regimens, called reduced intensity or non-myeloablative regimens, are meant to result in reversible myelosuppression and are often used for patients with significant organ dysfunction or recent severe infection before HCT (30). These regimens consist of lower doses of chemotherapy and radiation but higher amounts of immunosuppressive drugs to prevent rejection of the new graft, so these patients may not become profoundly neutropenic (25, 31). For the first year after transplant, their immune deficit is tied to lymphopenia, and as with myeloablative regimens, may persist for up to a year after transplant. Figure 1 summarizes infection risk by the intensity of preparative regimen.

## Graft Source Types

Two main types of HCT exist—*allogeneic* and *autologous* (32). Allogeneic stem cells are those that are donated by a healthy family member or unrelated volunteer (32). Autologous stem cells are those collected from the recipient prior to preparative chemotherapy (33). Allogeneic stem cells provide the advantage of allowing for the administration of high-dose chemotherapy with the addition of foreign cells to destroy a recipient's underlying malignancy (11). In the setting of allogeneic HCT, the first-choice donor is usually a human leukocyte antigen (HLA) matched sibling donor (34). If there is not a matched sibling available, then a HLA matched unrelated volunteer donor would be utilized (34).

Donor stem cells are collected directly from the BM space in a procedure called a BM harvest (35). They may also be collected via peripheral blood after the donor is given a stem cell mobilizing agent to move stem cells into the periphery for collection (36). Additionally, umbilical cord blood (UCB) is a rich source for stem cells and is used as an HCT source (37). Umbilical cord blood units are stored in registries and are assessed for HLA compatibility before infusion (37).

Autologous grafts are often thought of as “rescue” stem cells (38). For certain types of solid tumors [especially neuroblastoma and central nervous system (CNS) tumors], high-dose myeloablative chemotherapy regimens are followed by infusion of the patient's *own* stem cells, termed a “rescue” procedure (39). “Rescue” stem cell infusion allows for the administration of essentially fatal doses of chemotherapy or radiation with limited periods of neutropenia (39). For these patients, the chemotherapy that precedes the autologous graft administration is what is vital in curing the underlying malignancy (39).

## Engraftment

Once a patient receives their new stem cells via a central venous catheter, the cells circulate and home to the BM cavity (40). Here, each stem cell either makes another stem cell or gradually matures to eventually make a blood cell (41). Once sufficient cells have been made, the patient has achieved engraftment (42). The period of recovery that follows is dictated by the wait for engraftment, or the new BM's ability to produce blood cells including neutrophils, red blood cells, and platelets (43). Neutrophils are key in protecting from infection and are by convention used as the time indicator for the establishment of the new BM (42). Once the neutrophil count is sustained at >500 cells/μl for 3 consecutive days, the donor stem cells are considered “engrafted,” dating back to the first day of the 3-day consecutive run (42). Day to engraftment varies but in general, peripheral blood transplant recipients engraft on average at 14 days after the transplant day (known as day 0), BM recipients on day 14–21 days, and umbilical cord recipients on day 21–28 (44–47). For patients who receive autologous transplants, the time to engraftment is dependent on preparative therapy and underlying disease and typically ranges from 10 to 21 days (48, 49). Patients who do not engraft by day +42 following transplant are likely not to engraft at all, so this time point is considered the definition of graft failure (50).

The period immediately following the completion of preparative therapy, but before neutrophil recovery, is a high-risk period for infections (51). Prolonged neutropenia, mucosal barrier breakdown, central line presence, regimen-related toxicity (lung, renal, gastrointestinal, hepatic, and/or cardiac toxicities among others), and the altered microbiome all contribute to infection risk (52). Cell count reaches a nadir between 5 and 7 days after the preparative therapy and is often the time point when a critical care provider may encounter an HCT patient in need of intensive care secondary to infectious etiology (51).

As oral or gastrointestinal mucositis evolves, patients are at risk for infection by enteric Gram-negative bacilli, oral Gram-positive organisms, and gastrointestinal streptococcal species (21, 53). Septic shock in HCT patients can lead to high mortality rates, in particular when the originating bacterium is a Gram-negative organism (51). Patients remain at risk for infection with esophageal or enteric *Candida* species during this time (54). However, diagnosis of bloodstream yeast infections can be difficult to confirm despite frequent blood culturing. Sepsis from hepatosplenic or disseminated candidiasis can have delayed diagnosis and as a result poor response to therapy, once therapy is started (54). Sepsis from disseminated mold infections such as aspergillosis, mucormycosis, or fusariosis can carry mortality rates higher than 50% (55). Mucositis, combined with the absolute neutropenia and lymphopenia that can occur prior to engraftment, can lead to particularly severe reactivations of herpes simplex virus (HSV) oral infections, so patients often receive some form of viral prophylaxis (56). Viral reactivation by human herpesvirus 6 (HHV6) during neutropenia can lead to a fever of unknown origin that prolongs neutropenia (57).

The source of graft is an important factor in dictating this risk of infection, as each source is associated with a different length of time to engraftment and graduation from the most high-risk period for infection (42, 58). Prior studies have identified HCT using UCB as a donor source as a particularly high-risk graft due to delayed hematopoietic recovery and increased day 100 mortality (59). Additionally, Young et al. confirm that as peripheral blood transplants show the earliest engraftment, these patients often seem to suffer fewer infectious disease complications (9). Interestingly, when Barker et al. compared the infection risk in a cohort of pediatric patients who underwent HCT with unmanipulated BM or UCB graft, they found a comparable risk of bacterial and fungal infection between the two groups but higher risk of viral infection in the UCT group (60). This demonstrates that although each patient's risk factors must be taken into consideration, a general understanding of time to engraftment by graft source is a good estimator of the risk of infection.

## Graft-Vs.-Host-Disease

Graft-vs.-host-disease (GVHD) is a major cause of morbidity and mortality after allogeneic HCT, and a major risk factor for opportunistic infections (61). In general, the acute form of GVHD (aGVHD) occurs after engraftment within the first 100 days following HCT, while chronic GVHD occurs afterward (62). There may be some overlap between the acute and chronic forms of GVHD. Acute GVHD occurs when allogeneic transplanted cells recognize recipient antigens as foreign (63). Although there is much effort to match the best donor to the recipient based on HLA typing prior to transplantation, there may remain numerous minor antigens expressed by the recipient that are not tested for a match to the donor (64). Therefore, donor cells may attack or destroy recipient cells (65). Common anatomic sites for acute GVHD include the skin and liver, gastrointestinal tract (65). The etiology of chronic GVHD (cGVHD) is less understood than aGVHD but is due to T-lymphocyte imbalances and T-cell autoreactivity (66, 67). Most but not all patients who develop cGVHD have had aGVHD which can involve the skin, liver, eyes, and lungs among other organ systems (66).

Methods to decrease the risk of GVHD include graft manipulation and immunosuppressive medications (68, 69). T-cell depletion (TCD) of the BM or peripheral blood involves removing cyto-reactive T cells, after procurement of the graft (68). However, these same T cells are important for protection against bacterial, viral, and fungal infections (26). Van Burik et al. studied the development of infection by graft source in 404 adult patients and reported that the rate of infection did not differ between the TCD grafts and conventional GVHD prevention approaches; however, there was a greater incidence of severe CMV and aspergillosis in that patient cohort (70, 71). Again, an independent risk factor for infection was the development of severe acute GVHD (70). Therefore, for patients who receives a TCD graft, providers should be aware of these infectious risk factors.

Immunosuppressive medications given just prior to HCT and for months afterward is the main method to decrease the risk of GVHD (69). Active GVHD is treated with added immunosuppression, usually first with steroids and then with other stronger immune-suppressive drugs (67, 72). Immunosuppression to facilitate the acceptance of the new immune system and prevent the development of GVHD is given for 3–6 months following transplantation and then tapered off if no GVHD has developed (73). Both GVHD itself (by destroying the lymphoid microenvironment) and GVHD therapies (which are further immunosuppressive) place patients at risk for opportunistic infections (21, 74). Unlike solid organ transplant, however, allogeneic HCT patients do not require lifelong immunosuppression, as the recipient eventually develops tolerance of the donor cells (73). Since autologous transplant patients receive their own stem cells, they do not require immunosuppressive therapy after transplant (75).

## Immune Reconstitution

After neutrophil engraftment, the patient remains at extraordinarily high risk for bacterial, viral, and fungal infections since the immune system is still undergoing gradual reconstitution, which takes at least a year from transplant (76). It is important to understand that although engraftment is defined by neutrophil recovery, the components of the immune system are not entirely re-established as impaired cellular and humoral immunity persists (77). Neutrophils, monocytes, and NK cells are the initial cells that recover, followed by red cell and platelet recovery (78). Immune suppression delays this process further (78). Generally, patients receive immune suppression for 3–6 months after allogeneic HCT (69). For patients who develop aGVHD or cGHVD, immune suppression needs to be continued longer, often for years (67).

After engraftment but prior to full immune system reconstitution, impaired opsonization of encapsulated bacteria and central venous line presence all remain important risk factors for infections (76).

After engraftment, when there is the persistence of functional lymphopenia, the frequency of viral infections increases (79). Improved lymphopenia and T-cell competence do not occur until at least a year after HCT (79). Thus, this period places patients at risk for cytomegalovirus (CMV) viremia, varicella-zoster virus (VZV) shingles infections, and post-transplant lymphoproliferative disorder (PTLD) from Epstein Barr virus (EBV) (79). Although bacterial bloodstream infection remains the leading cause of organ failure, viral infections can be associated with high rates of mortality due to T-cell immune incompetence, which can persist for weeks to months following transplantation (5, 53). Bacterial infections are not uncommon but are more frequent in the early pre-engraftment phase and may be decreased with the use of prophylactic antibiotics (8).

## Surveillance Infectious Disease Testing

To understand a patient's infection risk prior to HCT extensive infectious disease testing is performed prior to the onset of preparative therapy (80). This includes testing of both the donor and the recipient (80). Test results from the recipient indicate whether any infection already exists that may pose a risk for reactivation after transplant (80). Positive test results from the donor that are discordant (negative) with the donor indicate whether the recipient is at risk for a primary viral infection acquired from the donor after transplant (81).

Tests include screening for previously acquired viral infections, including hepatitis B, hepatitis C, human immunodeficiency virus (HIV), human T-cell lymphotropic virus type 1 (HTLV), West Nile virus, syphilis, *Trypanosoma cruzi*, and since 2020, coronavirus disease 2019 (Covid-19) (21). All patients also undergo viral serology testing of HSV, VZV, EBV, and CMV to understand the possibility of reactivation during periods of immunosuppression (80). Active CMV infections, in particular, are known to portent poor outcomes after transplant and thus must be treated prior to proceeding with HCT (82). Barker et al. noted that positive recipient CMV status prior to transplant was associated with a 1.3 higher fold risk of serious infection, in a pediatric patient cohort of 136 patients (60).

Transplant candidates who live in or have spent time in high-risk areas will have extra testing prior to transplant (83). Depending on the specific geographic risks of an individual patient, they may be screened for endemic pathogens such as *Strongyloides stercoralis, Coccidioides* species, *Histoplasma capsulatum, Toxoplasma gondii*, and malaria (81).

Donor serology testing prior to transplant parallels candidate testing (21). Geographic screening is similar to that of the recipient and is inclusive of the infectious disease assessment for the expected recipient (83). Donor screening, depending on the type of graft, must be completed at least 7–30 days prior to stem cell collection. Screening includes both lab work and medical history with travel history noted (21). Infections such as HIV, acute CMV, acute hepatitis A, untreated active or latent tuberculosis, toxoplasmosis, and Zika virus are considered contraindications to donation (81). There may also be other infections that will need to be considered and tested for on a case-by-case basis (21). Most testing guidelines mirror blood donation standards (21).

Prior to HCT, patients also undergo radiological testing for evidence of any infection which may include computed tomography (CT) imaging of their chest and sinuses to screen for occult infection (84). For patients who have findings of infection by CT chest or sinus, transplant is delayed for diagnostic testing and/or anti-microbial treatment, with the goal of control or resolution of the infectious disease prior to initiating preparative therapy (85). It is known that patients with any active infection at the time of transplant have poorer outcomes, as the infection will likely worsen during the neutropenic phase of the transplant course (86).

Prior to the availability of azoles, proceeding with transplant with a known active fungal infection such as invasive aspergillosis or disseminated candidiasis led to poor outcomes and was widely considered a contraindication to HCT (87). Now, patients achieve comparable overall survival, non-relapse mortality, and relapse-free survival with appropriate pre-transplant antifungal treatment (88). Therefore, a history of active fungal infection prior to transplant is now tolerated during the pre-transplant assessment period, and patients may proceed into transplant after successful treatment with at least 4 weeks of anti-fungal therapy and radiographic resolution (or in some cases stability) of the infectious infiltrate (89). Some of these patients go on to receive additional antifungal medications during transplant (87).

Patients undergo surveillance diagnostic testing for certain viral infections (CMV, EBV, adenovirus) throughout the HCT process, with the most frequent surveillance occurring approximately weekly prior to the 100-day mark (90). Among those three viral infections, CMV is particularly known to present as a late-onset viral infection (91).

**Figure 2:**
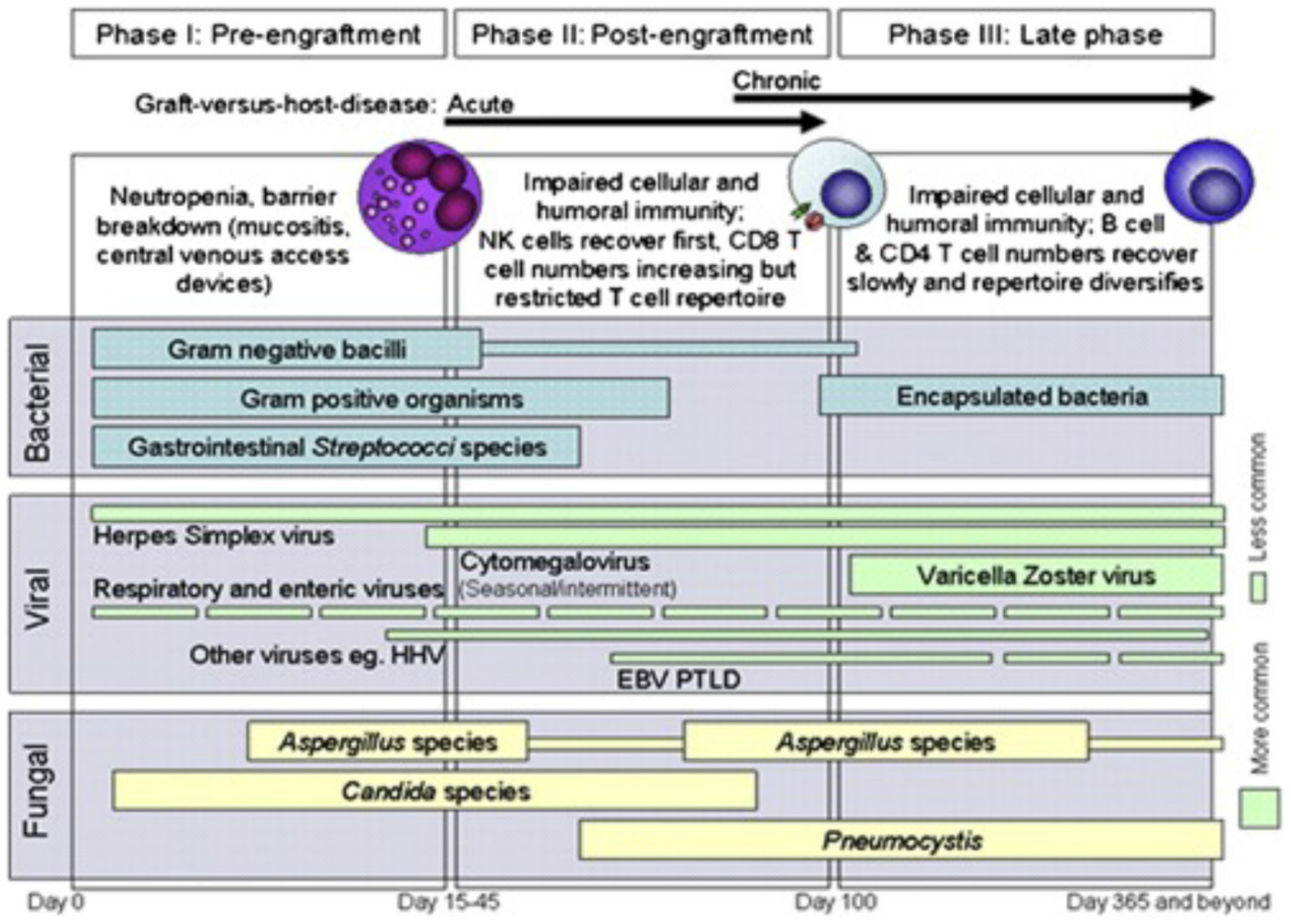
Phases of opportunistic infections among allogeneic HCT recipients. EBV, Epstein-Barr virus; HHV6, human herpesvirus 6; PTLD, post-transplant lymphoproliferative disease. Reprinted from Tomblyn et al. (21). Copyright 2009 by Elsevier, Reprinted with permission.

Figure 2 highlights how factors associated with HCT contribute to infection risk.

## Prophylactic Antimicrobial Use

Antimicrobial prophylaxis is important to prevent opportunistic infections during HCT (92). Based on a patient's pre-transplant risk characteristics, prophylactic anti-infective agents are initiated with the start of the preparative regimen and may include antibacterial, antiviral, and/or antifungal prophylaxis (10, 29).

In pediatric centers, guidelines for which bacterial agents to use for prophylaxis differ, but as a general rule HCT patients begin a fluoroquinolone a few days before HCT and continue the fluoroquinolone until neutrophils recover to a sustained value of >500 cells/μl (93–95).

Fungal infections (particularly *Aspergillus* species) can occur at any time during the transplant process but are often associated with prolonged neutropenia and particularly corticosteroid use (53, 96). However, many anti-fungal azole agents cannot be dosed during the chemotherapy portion of preparative therapy due to risks of potentially fatal drug–drug interactions and unacceptable hepatotoxicity (97). Therefore, patients often are placed on a broad spectrum echinocandin until chemotherapy completes, after which patients resume their prior prophylactic antifungal of choice given their transplant characteristics (96, 98).

Antiviral prophylaxis is used for patients at risk for CMV reactivation (transplant recipient is seropositive) or primary infection (donor is seropositive when the recipient is seronegative for prior infection) (99). Most centers utilize acyclovir or valacyclovir for this purpose (74). Adult patients receive letermovir in some centers, and this therapy may or may not cross over into pediatric transplant in coming years (100). Finally, patients remain on *Pneumocystis jirovecii* pneumonia (PJP) prophylaxis until 1-year following transplantation as the T-cell repertoire remains restricted (101).

## The Gastrointestinal Microbiome

Antimicrobial resistance and antibiotic stewardship are important facets to providing care for HCT patients (102). Due to preparative chemotherapy, mucosal damage, and frequent administration of empiric antimicrobials, the process of HCT is associated with severe intestinal dysbiosis (103). The rich diversity of the gut microbiome is disrupted and this process may allow for a single, often resistant, strain of bacteria to dominate (103). This loss of diversity is associated with increased overall mortality (104). The predominant mechanism for bacteremia following HCT is translocation of bacteria from the oral or gastrointestinal tract (105). Vancomycin-resistant *Enterococcus* and gram-negative bacteria can be associated with significant morbidity (105). As such, it is often routine practice to obtain weekly VRE surveillance rectal swabs for HCT recipients not known to be colonized with VRE (106, 107). Subsequently, for patients with known VRE colonization, it is imperative to consider whether to initiate VRE-active agents such as linezolid or daptomycin with fevers (108). Balancing successful prophylactic antimicrobial use with maintenance of the gastrointestinal microbiome is a continuous effort for transplant providers. An important future direction includes expanding understanding of other organ specific microbiomes including the oropharyngeal, dental, skin, and lung microbiomes.

## Vaccination After Transplantation

After HCT, patients do not have appropriate humoral or cell-mediated responses to immune insults (109). It is challenging to ensure protective immunity during this period of profound immunosuppression (110). Although ensuring adequate vaccination prior to transplant is helpful, all HCT patients require revaccination after transplant (109). If the HCT patient is receiving routine childhood vaccinations prior to HCT, they can receive inactivated vaccines no sooner than 2 weeks prior to preparative therapy and live vaccines no sooner than 4 weeks prior to preparative therapy (109).

Patients require reimmunization after HCT, which generally begins 1 year after HCT at which point their immune system has reconstituted sufficiently to ensure an adequate response to the vaccine (110). A notable exception is the non-live influenza vaccine which is given on day 60 or later after HCT (111). Vaccines given at 1 year all contain inactive organisms or parts of organisms (110). Live vaccines are administered 2 years after HCT to prevent uncontrolled proliferation of attenuated viral strains, as long as the patient is no longer receiving immune suppression (112). Serologic testing can be performed before measles, rubella, and varicella re-vaccination (113). The Bacillus Calmette-Guerin tuberculosis vaccine, oral poliovirus vaccine, cholera vaccine, oral typhoid vaccine, live zoster vaccine, yellow fever vaccine, and oral rotavirus vaccine are all contraindicated (114).

Vaccines may be given to those with GVHD (115). For patients who receive rituximab as part of their preparative therapy, B-cell recovery does not occur until 6–9 months after HCT (115).

Many providers may choose to trend the absolute CD4 and/or CD19 count as a surrogate marker for signs of T and B cell recovery, respectively (109). Often, vaccinations are deferred until the CD4 count is >200/μl and the CD19 count is >20/μl, although the use of these tests is highly variable to guide vaccination time points and can be center specific (116).

As is evident, there is yet much to be learned about optimizing vaccination schedule and discovering new markers of immune response. For the PICU provider, knowing that the HCT recipient is more than 2 years out from transplant suggests that their vaccine schedule may be up to date, and glancing at their immunization history could confirm that. Knowing that the HCT recipient is between 1 and 2 years from transplant suggests the patient may have received non-live vaccines. For the HCT patient who is not yet 1 year from the transplant procedure, the current illness may include infections with otherwise vaccine-preventable encapsulated organisms.

## Clinical Case Examples

**Figure 3:**
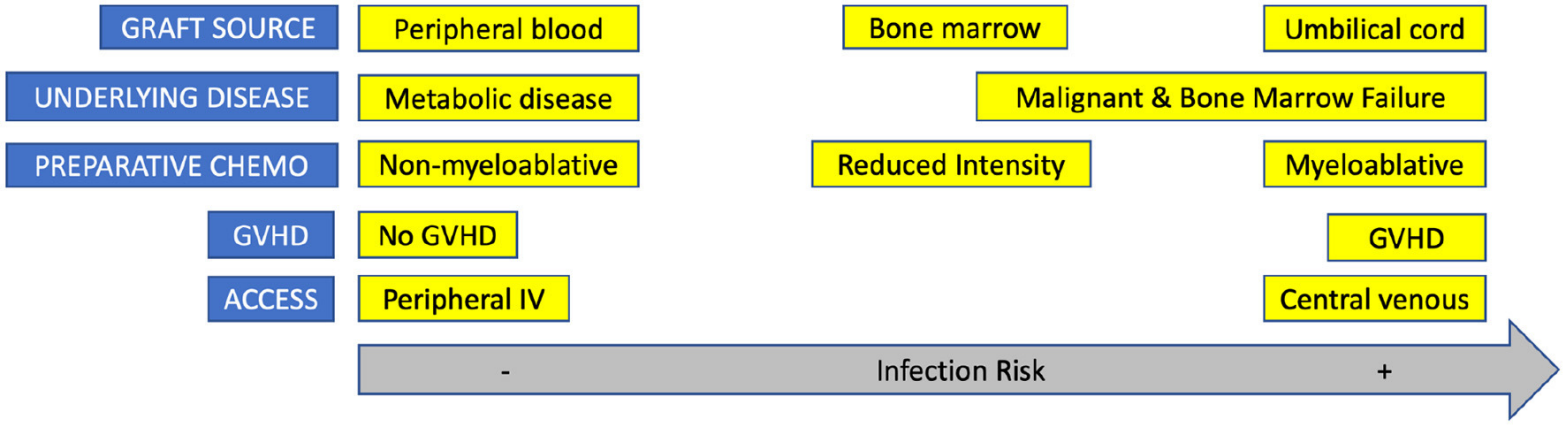
HCT characteristics and risk for infection.

It can be challenging to understand the exquisite state of an HCT patient's immune system during times of severe infections requiring PICU care. The important phases of HCT to consider when assessing a patient's infection risk and status are categorized into pre-preparative therapy, during preparative therapy, before engraftment, and after engraftment phases. Figure 3 highlights the shifting infectious risk of the HCT patient based on a variety of characteristics. We present a case-based overview and review of the management of the pediatric HCT recipient in the PICU with infectious complications by the phase of HCT. As immunosuppression plays a special role throughout HCT, it is discussed after the case vignettes. The following seven hypothetical cases are presented in order along the timeline for transplant.

### Case Presentation 1, Prior to Preparative Therapy for Transplant

A 2-year-old boy with a history of severe aplastic anemia (SAA) is undergoing pre-transplant infectious surveillance in anticipation of a myeloablative BMT. He initially presented with autoimmune hemolytic anemia (AIHA) and immune thrombocytopenia (ITP) following a viral illness and was treated for AIHA and ITP with intravenous IVIG, rituximab, and steroids. Due to the presence of autoimmune hepatitis, he was also being treated with azathioprine. His cytopenias progressed despite therapy over the next several months. He eventually developed pancytopenia leading to his diagnosis of SAA after BM biopsy showed cellularity of <5% (normal for age 90–95%). Pre-transplant infectious disease surveillance was unremarkable except for an abnormal chest CT. A 2.1 × 1.5 cm round subpleural mass was noted in the left lower lobe with ground-glass attenuation in the left perihilar region. On physical examination, the patient was afebrile, breathing comfortably in room air with a normal respiratory rate and oxygen saturation. **Question:** How should this patient be managed? **Answer:** HCT should be delayed while diagnostic procedures can be performed (bronchoscopy with bronchoalveolar lavage) and the patient receives applicable treatment for possible fungal pneumonia.

### Case Discussion

This patient has multiple risk factors for opportunistic and difficult-to-treat infections. First, his underlying diagnosis is aplastic anemia. Despite the misnomer, aplastic anemia is a failure of the BM to produce (or autoimmune destruction of) hematopoietic stem cells that later differentiate into leukocytes, red blood cells, and platelets. Therefore, depending on the timing of diagnosis, SAA patients may have spent weeks, months, or even years neutropenic by the time they come to HCT. The length of time between diagnosis and definitive treatment is related to survival outcomes (117). Additionally, the best mode of therapy in pediatric patients is to undergo a matched sibling HCT. In cases where a matched sibling is not available (75–80% of cases), patients are maintained on immunosuppressants typically consisting of anti-thymocyte globulin or cyclosporine—agents which can cause lymphopenia. This patient, who has a diagnosis of hepatitis-induced SAA, is also being treated with azathioprine which is immunosuppressive. Therefore, in this setting, this patient's diagnosis, as well as its subsequent treatment, are considered risk factors. This clinical vignette highlights the infectious risk factors of HCT patients even before they come to transplant, which reveals the risk factors inherent to the disease and its treatment. The provider needs to consider an HCT patient's risk factors that may exist outside the HCT process, which in and of itself is a risk factor.

### Case Presentation 2, During Preparative Therapy Prior to Transplant

An 8-year-old boy, with a history of relapsed acute myeloid leukemia (AML) after an UCT, is admitted to the hospital to begin preparation for a second HCT, using a second UCB graft that will be infused in 7 days. On the third day of his myeloablative preparative chemotherapy, 4 days prior to infusion of his second transplant, he develops a temperature of 39.8°C. On examination, he is hypotensive (blood pressure is 64/32 mmHg) and tachycardic at 150 beats/min. His lungs are clear to auscultation. Blood cultures are obtained from his central line, and he is started on broad-spectrum empiric antibacterial antibiotics. He receives multiple fluid boluses and his hemodynamic status improves. **Question:** What is the likely underlying etiology of his hemodynamic change? **Answer:** Gram-positive infection of his central venous catheter.

### Case Discussion

This patient is in the midst of his myeloablative preparative chemotherapy prior to his second transplant and is likely pancytopenic. This period marks the beginning of mucosal breakdown that can lead to opportunistic infections, as the preparative therapy affects rapidly dividing cells such as the mucosal surfaces. This effectively makes a patient's own commensurate bacteria a potentially fatal source for infection. In particular, oral streptococcal organisms can cause bloodstream and neck soft tissue infections during the first 3 weeks after transplant. As all patients undergoing HCT have a central venous access catheter throughout transplantation, it is important to consider the possibility of a catheter-associated infection with each fever, as described in the case presentation. All neutropenic HCT recipients who develop a fever are treated empirically with an antibiotic with anti-pseudomonal ± Gram-positive activity. In this setting, gram-positive bacteria are most commonly isolated. Coagulase-negative staphylococci may not be that virulent, but *Staphylococcus aureus* can be the cause of the severe hemodynamic collapse. Vancomycin is indicated in this setting and should be added to anti-pseudomonal therapy when there is an abrupt decline in hemodynamic status. For patients with central venous access catheters and hemodynamic stability, with cellulitis or pain around the indwelling catheter skin exit site, the addition of vancomycin is also appropriate.

### Case Presentation 3, Two Weeks After Transplant

A 6-year-old girl with a history of relapsed AML underwent a myeloablative matched sibling BMT 14 days ago. She is an inpatient in the hospital awaiting engraftment, receiving supportive care with a prophylactic fluoroquinolone antibiotic and transfusions until count recovery, when she suddenly develops fluid-refractory hypotension (blood pressure 70/30 mm Hg). On examination, she is noted to be febrile to 39.6°C and tachycardic at 145 beats/min. She is noted to have crackles on lung auscultation and has a distended but soft abdomen. She is urgently transferred to the PICU where she eventually requires support with multiple vasopressor agents and mechanical ventilation. She is started on broad-spectrum intravenous antibacterial antibiotics and undergoes CT imaging of her abdomen, which identifies colitis. **Question:** What is the likely underlying etiology of her sudden hemodynamic collapse? **Answer:** Gram-negative sepsis secondary to translocation of gut bacterial flora.

### Case Discussion

This patient developed rapidly deteriorating gram-negative septicemia. She received a matched sibling BMT 2 weeks ago and had likely not yet achieved engraftment. Therefore, she is at risk for severe opportunistic infections. For one, the gut microbiota are known to play a part in transplant-related infectious complications. She has also likely already experienced considerable mucositis and likely has some degree of epithelial cell damage in the gut which creates an environment in which commensal bacteria can invade the mucosa and submucosa to enter the bloodstream. Additionally, the preparative chemotherapy destroys circulating granulocytes and monocytes which are needed for gut healing (118). The colitis noted on the CT scan likely describes that mucosal injury with an associated influx of inflammatory mediators. During this phase of HCT where the patient has received preparative chemotherapy but has not engrafted their new cells, the provider must recognize the risk the patient is at from commensal aerobic and anaerobic bacterial organisms.

In the setting of an unidentified bacterial species or what becomes presumed culture-negative sepsis, many HCT recipients will receive double coverage with antibiotics directed at Gram-negative bacteria and anaerobic bacteria (119). An anti-pseudomonal beta-lactam can be escalated to a carbapenem (for anaerobic coverage). Additionally, an aminoglycoside (for gram-negative and some antipseudomonal coverage) is often initiated until hemodynamic stability is achieved and a causative organism identified. In the case of an unstable HCT patient, vancomycin can be added for better gram-positive gut flora (i.e., enterococcal) coverage, and anti-fungal coverage can be added or broadened to include coverage of regular candidal yeasts, fluconazole-resistant yeasts, and/or molds. As in this example, CT imaging of the abdomen is particularly important to perform and was concerning for neutropenic bacterial enterocolitis with possible intestinal necrosis.

### Case Presentation 4, One Month After Transplant

An 11-year-old boy with an immunodeficiency disorder underwent an HLA matched unrelated BMT. His transplant course was relatively uncomplicated and he had appropriate count recovery. On day 26 after his transplant, he developed high persistent fevers and a gradual change in mental status leading to florid encephalopathy. He was transferred to the PICU after experiencing a short tonic-clonic seizure and inability to maintain his airway. After intubation in the PICU, he underwent a lumbar puncture that revealed CSF findings of 5 WBC, 0 RBC, 96 mg/dl protein, and 47 mg/ml glucose (greater than two-third of the blood sugar level). CSF bacterial aerobic and anaerobic cultures remained negative. He was started on broad-spectrum antimicrobials including empiric antifungal and antiviral agents. **Question:** What viral infection is an emerging cause of encephalopathy in the HCT population? **Answer:** HHV6.

### Case Discussion

This case highlights the importance of maintaining a broad viral differential diagnosis, particularly for cases of encephalitis. HHV6 is a ubiquitous entity, with most individuals experiencing a primary infection in childhood and with some immunosuppressed individuals experiencing reactivation, as may be the case with HCT. HHV6 can remain latent in multiple organ systems including the CNS and in some cases may integrate its DNA into the human genome (120). Typical findings on MRI include limbic encephalitis but are not specific. The diagnosis is best made by an astute provider sending CSF DNA viral PCR studies (121). There remain no guidelines for the best course of therapy (122). Since HHV6 has ~75% DNA homology to CMV, various antiviral medications used to treat CMV infections can be used (123). It is important to note that the usual toxicities associated with use of these antiviral agents, such as BM suppression or kidney injury, can complicate treatment courses.

### Case Presentation 5, Six Months After Transplant

A 13-year-old girl with a history of relapsed acute lymphoblastic leukemia (ALL) underwent an UCT 6 months ago. She is engrafted without evidence of GVHD and is no longer receiving immunosuppression. She presents to the emergency department with a fever of 40.1°C and tachypnea. Her saturations are 88% while breathing room air so she is placed on low flow oxygen via nasal cannula. On examination, she is in obvious distress with increased work of breathing and decreased breath sounds in the left lower lung base with rhonchi. A chest radiograph is obtained which shows a large opacity in the left lower lung field with air-bronchograms and trace pleural effusion. **Question:** What vaccine-preventable infection should be considered? **Answer:** *Streptococcus pneumoniae*.

### Case Discussion

This patient was started on broad-spectrum antibacterial antimicrobial agents upon transfer to the PICU. A sputum sample was obtained which showed the predominant presence of gram-positive diplococci consistent with *S. pneumoniae*. A sputum culture confirmed the diagnosis. The differential diagnosis for bacterial causes for pneumonia in the immunocompromised HCT patients includes community-acquired bacteria such as *S. pneumoniae, Mycoplasma pneumoniae*, and *Haemophilus influenzae*. Influenza A and B viruses should be considered during annual flu seasons. Opportunistic fungi including *P. jirovecii, Aspergillus* spp., and *Cryptococcus* spp. Geographically-restricted fungi such as *Coccidioides immitis, Blastomyces dermatitidis*, and *H. capsulatum* should also be considered. Although not performed in this case, providers should consider bronchoalveolar lavage as a diagnostic tool for identifying either a community-acquired or opportunistic microorganism when sputum cultures do not lead to finding the reason for infection in the lungs (124–126).

### Case Presentation 6, 10 Months After Transplant

A 13-year-old boy with a history of SAA underwent a matched unrelated BMT 10 months ago. He is engrafted and has no history of GVHD. He is not receiving immunosuppressive therapy and remains on PJP prophylaxis per routine supportive care guidelines. He presents to his hematologist with a recent history of thrombocytopenia and a new-onset excruciating headache refractory to over the counter analgesia. He is admitted to the hospital for platelet transfusion and further imaging. Unfortunately, he experiences a sudden neurologic decline necessitating intubation. A brain MRI reveals numerous enhancing lesions throughout both cerebral and cerebellar hemispheres with punctate microhemorrhages and surrounding vasogenic edema. He undergoes extensive infectious disease testing of blood and cerebral spinal fluid. On lumbar puncture, he is noted to have elevated intracranial pressure (ICP) with a normal ophthalmologic exam. On expanded travel history, he is identified to have lived in China for multiple brief periods during his childhood, which assists in identifying his diagnosis. **Question:** What is the likely underlying infectious etiology of his MRI findings and neurologic decline? **Answer:** CNS Toxoplasmosis.

### Case Discussion

This patient presented with a CNS *T. gondii* infection, a somewhat unusual infection even in the heavily immunosuppressed BMT population and one that is often fatal. He was at risk for *Toxoplasma* infection because his *Pneumocystis* prophylaxis was changed from Bactrim to dapsone earlier in his post-transplant course. This case imparts an important lesson that non-sulfa-based Pneumocystis prophylaxis regimens do not protect against toxoplasmosis or nocardiosis or extrapulmonary pneumocystosis. Pneumocystis prophylaxis with inhaled pentamidine can lead to upper lung lobe Pneumocystis infections, where the nebulized pentamidine does not penetrate well.

For this case patient's infection, he was treated with a 6-week course of pyrimethamine, leucovorin, and sulfadiazine followed by maintenance pyrimethamine and clindamycin for 12 months. As *T. gondii* is known to have increased prevalence in geographical areas with cat exposure and possible food contamination with cysts, this case highlights the importance of soliciting a patient's full travel and pet exposure (127). It is important for the PICU provider to remain abreast of possible exposures a pediatric BMT patient may have and how that might cause systemic infection. Animals commonly kept as pets such as lizards, snakes, turtles, and other reptiles are known to carry and possibly spread *Salmonella* and *Cryptosporidium parvum* (128). Baby chicks and ducklings are known to carry and possibly spread *Campylobacter jejuni* (129). Cats are known to shed *T. gondii* in their feces as above and may carry *Bartonella henselae* (130). Finally, household dogs may be infected with parasites such as *Toxocara canis* (128).

### Case Presentation 7, 18 Months After Transplant

A 9-year-old boy with a history of cerebral adrenoleukodystrophy (c-ALD) underwent an UCT approximately 18 months ago. His HCT course was complicated by autoimmune cytopenias for which he received multiple therapies over the last year including rituximab, IVIG, mycophenolate mofetil (MMF), daratumumab, and steroids. He presented to the ED with increased work of breathing and oxygen saturation of 86% while breathing room air. On auscultation of his lungs, he had poor aeration bilaterally and copious coarse breath sounds. His Covid-19 testing was negative. He was transferred to the PICU for respiratory support. At the time of admission, he was not on any prophylactic antimicrobial agents. **Question:** What is the likely underlying etiology of his sudden respiratory distress? **Answer:** *Pneumocystis* pneumonia.

### Case Discussion

As this patient was over 1 year from HCT, PJP prophylaxis had been discontinued per standard procedure at the 1-year transplant clinic visit. However, he then developed autoimmune cytopenias and should have had *Pneumocystis* prophylaxis restarted when immunosuppressive treatments were resumed. In his case, he remained at risk for PJP while receiving highly immunosuppressive therapy for his autoimmune cytopenia and should have continued to receive prophylaxis due to continuing exogenous immunosuppression. It is important for providers to be aware that HCT patients must remain on PJP prophylaxis until 1 year after HCT or when patients are receiving >20 mg/day of steroid.

## Discussion

The PICU provider needs to understand the unique infectious risks present secondary to HCT and immunosuppression. Pediatric HCT patients who require PICU level care are complex and require a multisystem approach as well as a keen understanding of where they are in the transplant process. PICU providers must understand the shifting status of an HCT patient's infectious risk as they progress through the transplant process. Although immunosuppression is thought to be largely associated with the early phases of HCT, it is incumbent for the PICU provider to understand what if any immunosuppressive therapies the patient may still be receiving.

Respiratory and septic events are the main reasons HCT patients are transferred to the PICU. When evaluating these patients, it is important to also consider additional risk factors such as the presence of a central venous access catheter and the patient's time spent neutropenic and/or immunosuppressed. The care of the HCT recipient must remain focused on the likely multiorgan dysfunction that is present during times of infection. Although the clinical stability of a patient's hemodynamic status is paramount to good outcomes, providers must also consider antimicrobial stewardship. It is important to narrow antimicrobial coverage as soon as it is safe to do so (131). Use of too much antibacterial prophylaxis during neutropenia can have the deleterious effect of changing a patient's enteral microbiome (103). Pharmacists and infectious disease physicians alongside both the HCT and PICU physicians are key in maintaining this initiative. We hope this guide services as a reference for these patients when they require PICU level care.

## Author Contributions

All authors listed have made a substantial, direct and intellectual contribution to the work, and approved it for publication.

## Conflict of Interest

The authors declare that the research was conducted in the absence of any commercial or financial relationships that could be construed as a potential conflict of interest.

## Publisher's Note

All claims expressed in this article are solely those of the authors and do not necessarily represent those of their affiliated organizations, or those of the publisher, the editors and the reviewers. Any product that may be evaluated in this article, or claim that may be made by its manufacturer, is not guaranteed or endorsed by the publisher.
